# Autoinhibition of the mechanosensitive lipid scramblase TMEM63B by its C-terminal tail

**DOI:** 10.1016/j.jbc.2026.113223

**Published:** 2026-06-04

**Authors:** Megumi Nishimura, Yugo Miyata, Yu Shiraki, Risa Kuribayashi, Norimichi Nomura, Tomohiro Nishizawa, Katsumori Segawa

**Affiliations:** 1 Department of Medical Chemistry, Medical Research Laboratory, Institute of Integrated Research, Institute of Science Tokyo, Tokyo, Japan; 2 Graduate School of Medicine, Kyoto University, Kyoto, Kyoto, Japan; 3 Graduate School of Biostudies, Kyoto University, Kyoto, Kyoto, Japan; 4 Graduate School of Medical Life Science, Yokohama City University, Yokohama, Kanagawa, Japan; 5 Suntory Rising Stars Encouragement Program in Life Sciences (SunRiSE), Suntory Foundation for Life Sciences, Kyoto, Kyoto, Japan

**Keywords:** TMEM63B, OSCA/TMEM63 family, mechanosensitive ion channel, phospholipid scramblase, C-terminal tail, conformational regulation

## Abstract

TMEM63B belongs to the OSCA/TMEM63 family of mechanosensitive ion channels. We recently identified it as a mechanosensitive lipid scramblase activated by changes in membrane physical properties. Cryo-EM analysis revealed that recombinant mouse TMEM63B (mTMEM63B) protein adopts either closed or open conformations depending on the detergent environment, and that the monoclonal antibody YN9303-24 stabilizes the open state; however, the antibody epitope and the mechanism of antibody-dependent conformational regulation remained unclear. Here, using chimeric constructs, C-terminal truncations, and internal deletions, we mapped the YN9303-24 epitope to the intracellular C-terminal tail and identified the AQV motif (residues 773–775) as the core binding determinant. Functional analyses revealed that this C-terminal region is essential for maintaining TMEM63B in an inactive state under resting conditions. Deletion of the adjacent LQD motif (Δ776–778) or substitution of Leu776 with alanine induced strong constitutive lipid scrambling, evidenced by phosphatidylserine externalization and enhanced incorporation of fluorescently labeled phosphatidylcholine, whereas substitutions at Gln777 or Asp778 had minimal effects. Structural analysis positioned the AQVLQD motif adjacent to conserved intracellular helices in the open conformation, with Leu776 located near several hydrophobic residues. Together, these findings identify an autoinhibitory role for the C-terminal tail region in maintaining TMEM63B in an inactive state, suggesting that interactions between this tail and intracellular helices regulate the activity of this mechanosensitive lipid scramblase.

Biological membranes exhibit a tightly regulated phospholipid asymmetry. In the plasma membrane (PM), aminophospholipids such as phosphatidylserine (PS) and phosphatidylethanolamine are predominantly confined to the cytoplasmic leaflet, whereas phosphatidylcholine (PC) and sphingomyelin (SM) are enriched in the exoplasmic leaflet (1, 2). This asymmetry is maintained by ATP-dependent flippases, primary type IV P-type ATPases (3, 4, 5, 6), and can be rapidly dissipated by ATP-independent lipid scramblases that catalyze bidirectional phospholipid translocation across the bilayer (7). Controlled loss of asymmetry underlies diverse processes, including apoptotic cell clearance, blood coagulation, myoblast fusion, and microvesicle release (8). Two major scramblase families have been identified in mammals: XKR8 proteins, which are activated by caspase-mediated cleavage during apoptosis, and TMEM16 proteins, some of which directly bind Ca^2+^ and function as Ca^2+^-activated lipid scramblases (7). Structural and functional studies of TMEM16 scramblases have revealed a membrane-spanning hydrophilic groove that supports a “credit-card”–like pathway through which phospholipid headgroups traverse the lipid bilayer (7, 9).

TMEM16 proteins share structural similarity with the OSCA/TMEM63 mechanosensitive ion channels and with the TMC (transmembrane channel-like) family (10). Plant OSCA channels assemble as dimers, with each subunit harboring an ion-conducting pore (11, 12, 13), whereas recent cryo-EM structures show that mammalian TMEM63A-C proteins are monomeric and comprise eleven transmembrane helices that form a membrane-embedded cavity (14). OSCA/TMEM63 and TMC proteins appear to possess lipid-accessible or lipid-lined permeation pathways, and for OSCA channels, a belt of lipids bridging the two membrane leaflets has been proposed to participate in ion conduction (13, 14). We and others have reported that TMEM63B exhibits lipid scramblase activity in both cell-based assays and cell-free systems using recombinant protein (15, 16, 17, 18). We showed that mammalian TMEM63B scramblase activity is induced by methyl β-cyclodextrin (MβCD) and phospholipase treatments that generate compositional or qualitative imbalances between the two leaflets of the lipid bilayer, indicating that TMEM63B responds to changes in membrane physical properties (15, 16). Cryo-EM analyses are consistent with this notion: in LMNG–CHS detergent, mTMEM63B adopts a closed conformation, whereas in DDM–CHS it shifts to an open state only in the presence of the YN9303-24 monoclonal Ab (mAb) raised against mTMEM63B (15). This reflects an intrinsic sensitivity of TMEM63B to its membrane environment, with YN9303-24 stabilizing the open state, allowing us to identify its lipid translocation cavity formed by transmembrane segments (TM) 4–6 (15). However, because Ab density is not visible in the cryo-EM map, the precise epitope and the mechanism by which YN9303-24 promotes channel opening in DDM–CHS remain unknown.

In this study, we mapped the YN9303-24 mAb epitope to the C-terminal tail of mTMEM63B and identified the AQV motif at residues 773–775 as its core determinant. We further show that this C-terminal region is functionally important: deletion of the neighboring LQD motif or substitution of Leu776 with alanine converts TMEM63B into a constitutively active lipid scramblase. Together, these results indicate that the C-terminal tail is required for maintaining TMEM63B in its inactive form under steady-state conditions.

## Results

### Identification of the YN9303-24 mAb epitope

**Figure 1:**
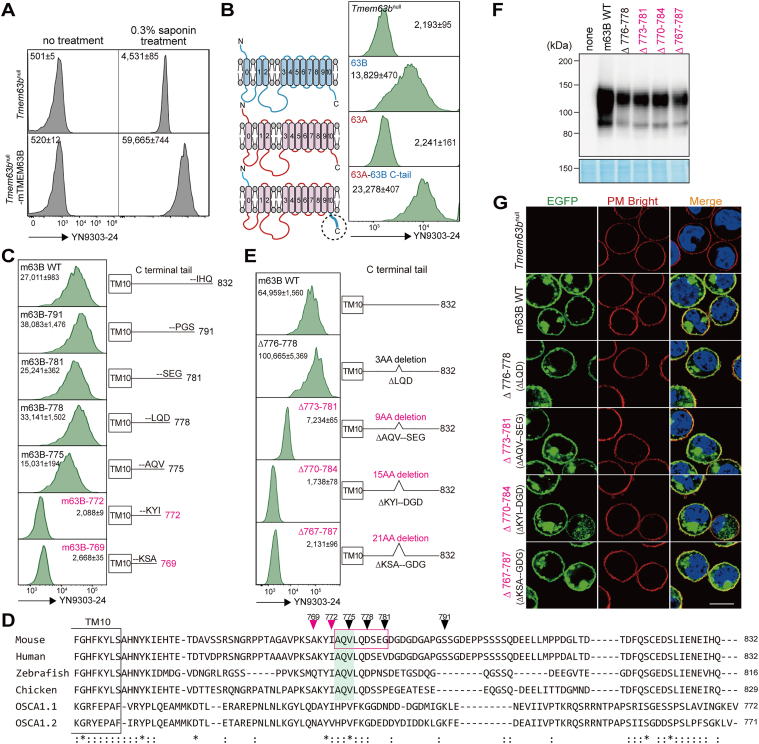
**Identification of the YN9303-24 epitope in the C-terminal tail of mTMEM63B.***A–C*, and *E*, the indicated cells were incubated with or without 0.3% saponin, stained with 1 μg/ml YN9303-24 mAb, and analyzed by flow cytometry. Representative histograms are shown for *Tmem63b*^null^ cells expressing wild-type (WT) mTMEM63B (*A*); hTMEM63B, hTMEM63A, and a hTMEM63A–B chimera in which the N- and C-terminal tails were swapped with those of hTMEM63B (*B*); serial C-terminal truncations of mTMEM63B (*C*); and mTMEM63B mutants with the indicated internal deletions (*E*). Data are from three independent experiments; mean ± S.D. of median fluorescence intensity (MFI) is shown. Mutants that lost YN9303-24 binding are highlighted in magenta. *D*, Alignment of the C-terminal amino acid sequences of TMEM63B orthologs. Sequences of the orthologs from the indicated species (mouse: NP_937810.2, human: NP_001305721.1, zebrafish: XP_005157122.1, chicken: NP_001366170.1, OSCA1.1: NP_849297.1, and OSCA1.2: NP_001078425.1) were aligned using Clustal Omega. Transmembrane segment 10 (TM10) is boxed. Positions of the serial C-terminal truncations of mTMEM63B are indicated by arrowheads. The amino acids at positions 773–781 are boxed in *magenta*. Conserved residues and residues with high similarity are indicated by *asterisks* and *colons*, respectively. The center of the epitope, residues 773–775 (AQV), is highlighted in *green*. *F*, *top*, cell lysates of *Tmem63b*^null^ cells expressing EGFP-tagged WT mTMEM63B or mTMEM63B mutants were analyzed by Western blotting with anti-GFP Ab. *Bottom*, CBB staining of the membrane as a loading control. *G*, the indicated cells were analyzed by confocal microscopy in the presence of PlasMem Bright (*red*). Merged images of EGFP (*green*), PlasMem Bright, and Hoechst 33342 (*blue*) are shown. Scale bar, 10 μm.

As reported (15), YN9303-24 bound to mouse (m) TMEM63B-expressing *Tmem63b*^null^ Ba/F3 cells (a mouse pro-B lymphoma line) only after saponin-mediated permeabilization, but not to *Tmem63b*^null^ cells (Fig. 1*A*), indicating that the epitope is located in an intracellular region. mTMEM63B shares 98.2% and 54.2% amino acid identity with human (h) TMEM63B and hTMEM63A, respectively. Consistent with this, YN9303-24 bound hTMEM63B but not hTMEM63A (Fig. 1*B*), suggesting that hTMEM63A lacks the relevant epitope. TMEM63B has three major intracellular regions: the short segment between TM0 and TM1, the large internal segment between TM2 and TM3, and the C-terminal tail. We focused on the C-terminal tail as a candidate epitope because protein termini are typically flexible, solvent-exposed, and frequently serve as linear B-cell epitopes (19). In line with this, swapping the N- and C-terminal regions of hTMEM63A with those of hTMEM63B conferred YN9303-24 binding on hTMEM63A after saponin treatment (Fig. 1*B*), indicating that the intracellular C-terminal tail of TMEM63B plays a critical role in Ab recognition.

To further investigate the role of the C-terminal tail in mAb binding, we stably expressed a series of C-terminal truncations of EGFP-tagged mTMEM63B in *Tmem63b*^null^ cells and examined their binding to YN9303-24. Deletion of the C-terminal tail slightly reduced protein expression as detected by Western blotting with an anti-GFP Ab, but PM localization was preserved, indicating that expression, folding, and targeting were maintained (Fig. S1, *A* and *B*). As shown in Figure 1*C*, YN9303-24 robustly bound cells expressing WT mTMEM63B or C-terminal truncates deleted up to residue 778 (m63B-791, m63B-781, and m63B-778), whereas extending the deletion to residue 775 (m63B-775) reduced binding by ∼50%, and further deletion abolished binding completely. These results indicate that residues 773–778 (AQVLQD) contribute to YN9303-24 recognition, with the AQV residues at positions 773–775 serving as the critical determinant (Fig. 1, *C* and *D*). We next examined mutants carrying a series of internal deletions within this region. As shown in Figure 1*E*, YN9303-24 bound cells expressing the Δ776–778 mutant with a three–amino acid deletion (LQD) similarly to WT mTMEM63B, whereas cells expressing the Δ773–781 mutant with a nine–residue deletion (AQVLQDSEG) or mutants with larger deletions (Δ770–784 and Δ767–787) showed negligible binding. All mTMEM63B mutants with internal deletions were expressed and localized to the PM (Fig. 1, *F* and *G*). Together, these data indicate that the three residues AQV at positions 773–775 constitute the core of the YN9303-24 epitope.

### Critical role of the C-terminal tail in maintaining the inactive state of TMEM63B lipid scramblase

**Figure 2:**
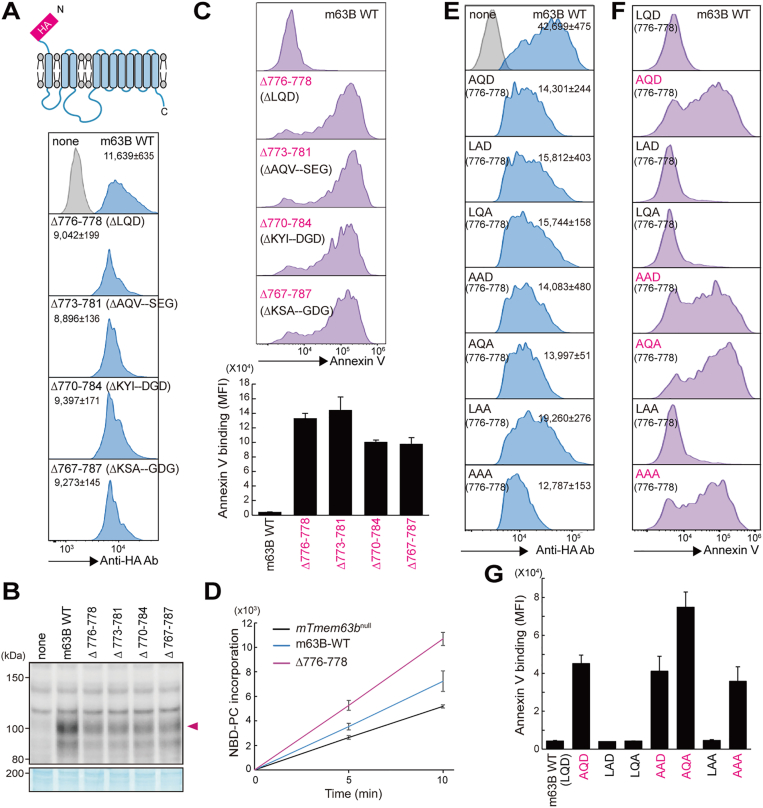
**Constitutive lipid scrambling caused by a point mutation at Leu776 in the C-terminal tail of mTMEM63B.***A* and *E*, *Tmem63b*^null^ cells (*gray*) or *Tmem63b*^null^ cells expressing the indicated mTMEM63B WT or mutants (*blue*) were incubated with anti-HA Ab, and analyzed by flow cytometry in the presence of SYTOX Blue to exclude dead cells. Representative histograms of anti-HA staining in SYTOX Blue–negative populations are shown. Experiments were repeated three times; mean ± S.D. of median fluorescence intensity (MFI) is indicated in each histogram. *B*, cell lysates of *Tmem63b*^null^ cells or *Tmem63b*^null^ cells expressing mTMEM63B mutants with the indicated internal deletions were analyzed by Western blotting with anti-HA Ab. *Bottom*, CBB staining of the membrane as a loading control. *C*, *F*, and *G*, *Tmem63b*^null^ cells expressing the indicated mTMEM63B WT or mutants were incubated with Annexin V and analyzed by flow cytometry. *C* and *F*, representative histograms of Annexin V staining in SYTOX Blue–negative populations are shown. Experiments were repeated three times; mean ± S.D. of MFI is shown (*G*). Mutants with constitutive PS scrambling activity are highlighted in *magenta*. *D*, *Tmem63b*^null^ cells or *Tmem63b*^null^ cells expressing WT mTMEM63B or the Δ776–778 mutant were incubated with NBD-PC at 15 °C and analyzed by flow cytometry. Experiments were repeated three times; mean ± S.D. of MFI is shown.

Ba/F3 cells express TMEM63A and TMEM63B, but not TMEM63C, among the TMEM63 paralogs (Fig. S1*C*). Of the TMEM63 family members, only TMEM63B exhibits mechanosensitive lipid scramblase activity at the PM; that is, Ba/F3 cells expressing m/hTMEM63B expose PS and incorporate fluorescently labeled PC or SM at the cell surface when membrane thickness or curvature is altered by MβCD or phospholipases (15, 16). We previously showed that YN9303-24 mAb stabilizes or promotes an open conformation of recombinant mTMEM63B under DDM–CHS conditions (15), suggesting that the C-terminal epitope region can affect gating of the lipid translocation pathway. To test this, we generated HA-tagged mTMEM63B mutants lacking residues around the YN9303-24 epitope and expressed them in *Tmem63b*^null^ cells. The HA tag was placed at the extracellular N terminus to monitor surface expression and protein topology. Without permeabilization, all the mutants were detected on the cell surface by anti-HA Ab staining at 70–75% of WT levels (Fig. 2*A*), and this was also confirmed by Western blotting (Fig. 2*B*). Despite slightly decreased surface expression, cells expressing the Δ776–778 mutant lacking the LQD motif showed strong constitutive scramblase activity under steady-state conditions, as detected by intense Annexin V staining of exposed PS (Fig. 2*C*). Mutants with longer deletions around this motif behaved similarly, indicating that removal of the LQD segment confers constitutively active lipid scramblase activity on TMEM63B. Consistently, NBD-PC incorporation was also increased in cells expressing the Δ776–778 mutant, confirming enhanced bidirectional phospholipid scrambling (Fig. 2*D*). To pinpoint the contribution of individual residues, we introduced all possible alanine substitutions within the LQD motif in full-length HA-mTMEM63B and expressed these mutants in *Tmem63b*^null^ cells. All Ala-substitution mutants were present at the PM and detected on the cell surface by anti-HA Ab at 24–40% of WT levels (Fig. 2*E*). PS exposure assays revealed a specific requirement for Leu776 in the C-terminal tail (Fig. 2, *F* and *G*): any mutant carrying an Ala substitution at Leu776 displayed robust, constitutive PS exposure, whereas substitutions at Gln777 or Asp778 had little or no effect. These results identify Leu776 in the C-terminal tail as essential for stabilizing the inactive state of mTMEM63B under steady-state conditions.

## Discussion

**Figure 3:**
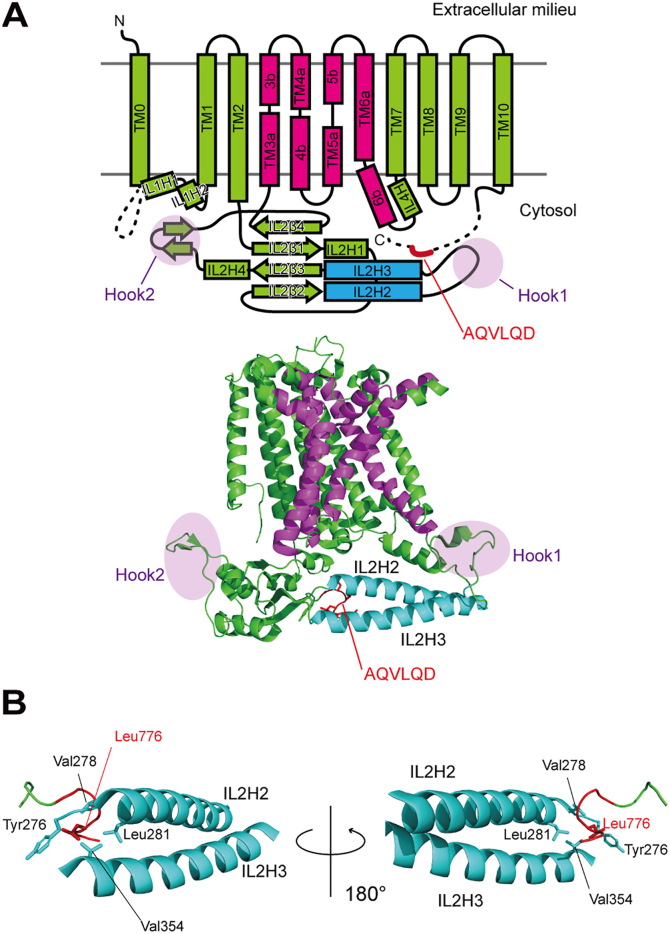
**Interaction between Leu776 in the C-terminal tail and two intracellular amphipathic helices.***A*, *top*, schematic of mTMEM63B (PDB: 8WG4; open mTMEM63B in DDM–CHS with YN9303-24 Ab). Transmembrane (TM) helices and intracellular linkers (ILs) with α-helix (H) and β-sheet (β) are shown with numbering. TM helices that form the lipid translocation cavity are colored in *magenta*. Palmitoylated hook regions required for TMEM63B scrambling are highlighted in *purple*. The AQVLQD motif is colored in *red*. Two intracellular amphipathic helices (IL2H2 and IL2H3) constitute a beam-like domain and contain the hook regions. The AQVLQD motif is located adjacent to these helices. *B*, hydrophobic residues in IL2H2 and IL2H3 positioned near Leu776 are shown.

mTMEM63B contains eleven transmembrane segments and a large intracellular segment with two long amphipathic helices (IL2H2 and IL2H3) between TM2 and TM3 that run parallel to the cytosolic membrane surface (Fig. 3*A*). These helices, conserved among TMEM63/OSCA family members, constitute a beam-like domain (14). We previously showed that the lipid translocation cavity is formed by TM3–TM6 and that palmitoylated intracellular hook regions connected to IL2H2 and IL2H3 are critical for membrane structure–responsive scramblase activity (15). Here, we identified the YN9303-24 mAb epitope as the AQV motif (residues 773–775) and demonstrate that the C-terminal tail, particularly Leu776 within the AQVLQD motif, is essential for maintaining mTMEM63B in its inactive state.

In the closed-state structure, density corresponding to the C-terminal tail, including the AQVLQD motif, is not resolved (15), indicating high flexibility in the inactive state. By contrast, in the DDM–CHS open-state structure with YN9303-24, density for the AQVLQD motif is observed adjacent to IL2H2 and IL2H3, with Leu776 surrounded by hydrophobic residues including Tyr276, Val278, and Leu281 in IL2H2 and Val354 in IL2H3 (Fig. 3, *A* and *B*), suggesting that this motif associates with the helices in the beam-like domain *via* hydrophobic interactions. Antibody binding appears to stabilize this conformation, and transient engagement of the AQVLQD motif with the helices helps maintain the inactive state. In this model, dynamic contacts between the flexible C-terminal tail and the beam-like domain stabilize the closed conformation at rest, whereas changes in membrane structure alter these interactions and allow opening of the lipid translocation pathway. The Leu776–beam-like domain interaction also appears critical for mTMEM63B expression (Fig. 2), suggesting that the C-terminal tail contacts with the intracellular helices also regulate protein folding. Collectively, the C-terminal tail intrinsically functions as an autoinhibitory element that maintains TMEM63B in an inactive state while also supporting protein folding. However, we cannot entirely rule out the possibility that the AQVLQD motif transiently interacts with other proteins, thereby modulating its contact with the beam-like domain. Niu *et al.* reported that mTMEM63B associates with mSLC19A2 (17), suggesting that additional proteins may interact with TMEM63B transiently or dynamically in cells. Key questions include how the C-terminal–beam-like domain interaction dynamically responds to changes in membrane structure and whether additional proteins participate in this process. High-resolution structures in native membrane environments will be needed to define the geometry and dynamics of C-terminal tail–mediated regulation.

## Experimental procedures

### Cell lines, plasmids, reagents, and antibodies

HEK293T cells (RRID: CVCL_0063) were originally obtained from ATCC and maintained in DMEM supplemented with 10% fetal bovine serum (FBS) at 37 °C in a 5% CO_2_ incubator. The mouse IL-3–dependent pro-B cell line Ba/F3 (RRID: CVCL_0161) was cultured in RPMI 1640 medium containing 10% FBS and 80 U/ml recombinant mouse IL-3 (20). *Tmem63b*-deficient Ba/F3 cells (*Tmem63b*^null^) were established as described (15). The pMXs-puro vector and pGag-pol-IRES-bsr packaging plasmid were provided by Dr T. Kitamura (Foundation for Biomedical Research and Innovation) (21), and pCMV-VSV-G was from Dr H. Miyoshi (Riken Bioresource Center). pAdVAntage was purchased from Thermo Fisher Scientific. cDNAs for hTMEM63A (NM_014698.3) and hTMEM63B (NM_001318792.1) were obtained from Dr M. McManus (#161667 and #161682, Addgene). mTMEM63B (NM_198167.4) was prepared as described (15, 16). An expression plasmid for the hTMEM63A/hTMEM63B chimera was constructed using seamless assembly. Briefly, the transmembrane and cytoplasmic regions of hTMEM63A and the C-terminal region of hTMEM63B were amplified by PCR. The resulting fragments were assembled into the PacI and EcoRI sites of the pMXs-puro vector containing a C-terminal EGFP tag using the NEBuilder HiFi DNA Assembly kit (NEB). C-terminal truncation mutants of mTMEM63B were generated by PCR amplification from WT mTMEM63B cDNA. These DNA fragments were inserted into either the EcoRI and FspAI sites of the pMXs-puro vector carrying a C-terminal EGFP tag or the PacI and NotI sites of the pMXs-puro-mCherry-P2A-HA vector using the NEBuilder HiFi DNA Assembly kit. DNA fragments carrying internal deletions within the C-terminal epitope region were generated by PCR and inserted into the pMXs vector as above. All plasmids were verified by Sanger sequencing. The primers used in this study are listed in Table S1.

The anti-mTMEM63B monoclonal Ab (mAb) YN9303-24 was generated and purified as described (15). A horseradish peroxidase (HRP)-conjugated rabbit anti-GFP polyclonal Ab (PM598-7) and an Alexa Fluor 488-conjugated mouse anti-HA mAb (M180-A48) were from Medical & Biological Laboratories (MBL). A mouse anti-HA mAb (901501) was from BioLegend. An Alexa Fluor 647-conjugated goat anti-mouse IgG (H + L) secondary Ab (A-21235) and an HRP-conjugated goat anti-mouse IgG polyclonal Ab (SA00001-1) were from Thermo Fisher Scientific and Proteintech, respectively. PVDF Blocking Reagent for Can Get Signal (NYPBR01), Can Get Signal Solution 1 (NKB-201), and Can Get Signal Solution 2 (NKB-301) were from TOYOBO. Hoechst 33342 (19172-51) and PlasMem Bright Red (P505) were from Nacalai Tesque and Dojindo, respectively. Annexin V-Cy5 (ab14147) was from Abcam. NBD-PC (810130) was from Avanti Polar Lipids. For real-time RT-PCR, the RNeasy Mini Kit (Qiagen, #74104), High-Capacity RNA-to-cDNA Kit (Thermo Fisher Scientific, #4387406), and SsoAdvanced Universal SYBR Green Supermix (Bio-Rad, #172-5271) were used.

### Retrovirus-mediated establishment of stable cell lines

The pMXs-puro vector carrying EGFP- or HA-tagged cDNA was transfected into HEK293 T cells using FuGENE6 (Promega) together with pGag-pol-IRES-bsr, pCMV-VSV-G, and pAdVAntage. At 48 h post-transfection, retroviral supernatants were filtered through a 0.45-μm membrane (Merck Millipore), concentrated using a Retro-X concentrator (TAKARA), and used to infect *Tmem63b*^null^ cells. Transformants were then selected with 1 μg/ml puromycin for 2 to 4 days. When necessary, cells expressing the introduced constructs were sorted using a CytoFLEX SRT (Beckman Coulter).

### Western blotting

Cells were lysed in RIPA buffer (50 mM HEPES-NaOH, pH 7.4, 150 mM NaCl, 1% Nonidet P-40, 0.1% SDS, and 0.5% sodium deoxycholate) containing a protease inhibitor cocktail (Nacalai Tesque). Protein concentrations were determined using a BCA assay (Nacalai Tesque). Lysates were mixed with SDS sample buffer (final concentrations: 68 mM Tris-HCl, pH 6.8, 2% SDS, 10% glycerol, 1% 2-mercaptoethanol, and 0.005% bromophenol blue) at RT for 20 min. Samples were separated by electrophoresis on 7.5% polyacrylamide gels (Nacalai Tesque) and transferred to PVDF membranes (Merck Millipore). For EGFP detection, membranes were blocked with 5% skim milk in TBS containing 0.05% Tween-20, followed by incubation with an HRP-conjugated anti-GFP Ab overnight at 4 °C. Chemiluminescent signals were detected using Chemi-Lumi One L (Nacalai Tesque) and imaged with a ChemiDoc Touch MP (Bio-Rad). For HA detection, membranes were blocked with PVDF Blocking Reagent for Can Get Signal, followed by incubation with an anti-HA Ab diluted in Can Get Signal Solution 1 overnight at 4 °C. Membranes were then incubated with an HRP-conjugated anti-mouse IgG Ab diluted in Can Get Signal Solution 2 for 1 h. Chemiluminescent signals were detected using Chemi-Lumi One Super (Nacalai Tesque) and imaged with a ChemiDoc Touch MP (Bio-Rad). After chemiluminescent detection, PVDF membranes were stained with PageBlue Protein Staining Solution (Thermo Fisher Scientific) to visualize total protein.

### Real-time RT-PCR

Total RNA was extracted from Ba/F3 cells using the RNeasy Mini Kit (Qiagen), reverse-transcribed into cDNA using the High-Capacity RNA-to-cDNA Kit (Thermo Fisher Scientific), and subjected to real-time PCR with SsoAdvanced Universal SYBR Green Supermix (Bio-Rad) on a CFX Duet Real-Time PCR System (Bio-Rad). Primer sequences are listed in Table S1.

### Ab binding

Cells were incubated in PBS containing 2% FBS, with or without 0.3% saponin, for 10 min at RT, then incubated with 0.5 μg/ml YN9303-24 for 20 min. After washing, cells were incubated with an Alexa Fluor 647-conjugated goat anti-mouse IgG Ab (0.5 μg/ml) at 4 °C for 30 min. Cells were washed with PBS containing 0.1% saponin and analyzed using a CytoFLEX S (Beckman Coulter). For detection of the N-terminal HA tag of mTMEM63B on the cell surface, cells were incubated under non-permeabilizing conditions with 10 μg/ml anti-HA Ab for 30 min at 4 °C, washed, and analyzed using a CytoFLEX S.

### Detection of PS on the cell surface and NBD-PC incorporation

To detect PS exposure, 3 × 10^5^ cells were suspended in Annexin V binding buffer (10 mM HEPES-KOH (pH 7.5), 140 mM NaCl, 2.5 mM CaCl_2_) containing Annexin V-Cy5 (1:1000 dilution) and incubated at 4 °C for 30 min. Cells were analyzed by CytoFLEX S in the presence of 1 μM SYTOX Blue (Thermo Fisher Scientific).

For NBD-PC incorporation, 5 × 10^5^ cells were suspended in HBSS containing 1 mM MgCl_2_ and 2 mM CaCl_2_ (HBSS^++^) and incubated at 15 °C for 7 min and then incubated with 10 nM NBD-PC in 500 μl of HBSS^++^ for the indicated times. A 100 μl aliquot of the cell suspension was mixed with 150 μl of HBSS containing 50 mg/ml fatty acid-free BSA (Merck) to extract unincorporated NBD-PC from the outer leaflet of the PM (22). Cells were also analyzed by CytoFLEX S in the presence of 1 μM SYTOX Blue.

### Confocal microscopy

Cells were incubated with 10 μg/ml Hoechst 33342 at RT for 5–10 min to visualize nuclei, followed by staining with 50 nM PlasMem Bright Red for 1 min to label the PM. The cell suspension was immediately transferred to a glass-bottom dish (Matsunami Glass) and observed using a confocal microscope (FV3000, Olympus). ImageJ was used for image processing.

## Data availability

The data that support the findings of this study are available from the corresponding author upon request.

## Supporting information

This article contains supporting information.

## Conflict of interest

The authors declare that they have no conflicts of interest with the contents of this article.
