# Supplementary material for: Autoinhibition of the mechanosensitive lipid scramblase TMEM63B by its C-terminal tail
Source: J Biol Chem. 2026 Jun 4;302(7):113223. doi: 10.1016/j.jbc.2026.113223 (PMC13332465; doi:10.1016/j.jbc.2026.113223)
Supplement: Figure S1 [file mmc2.pdf]

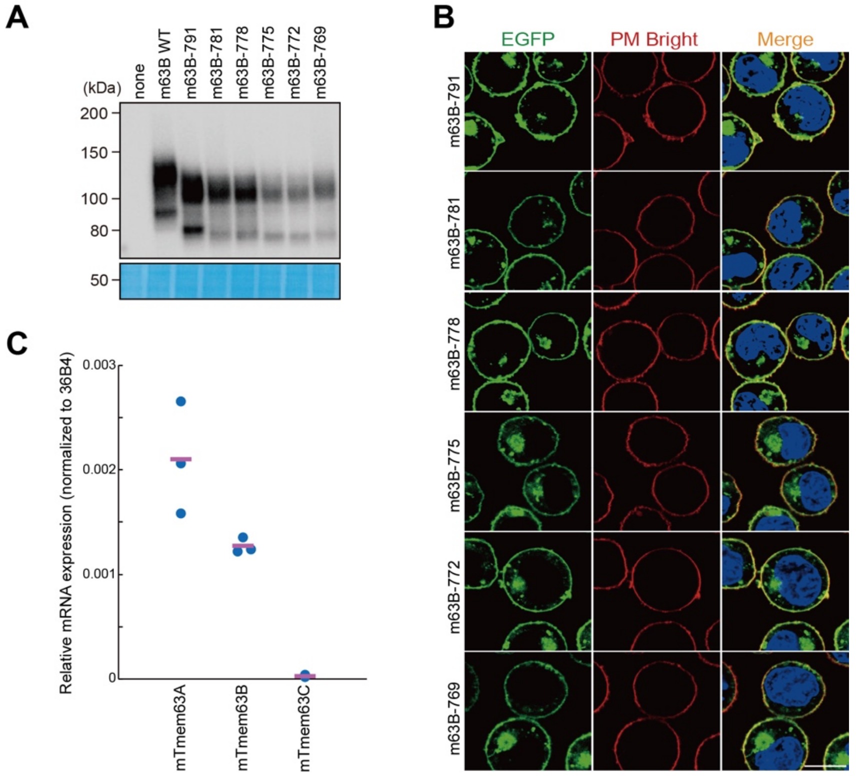

Fig. S1. Expression of mTMEM63B C-terminal truncates and mRNA levels of m*Tmem63* family members in Ba/F3 cells. (A) Cell lysates of *Tmem63b*^{null} cells or *Tmem63b*^{null} cells expressing EGFP-tagged WT mTMEM63B or mTMEM63B mutants with the indicated C-terminal deletions were analyzed by Western blotting with anti-GFP antibody. Bottom, CBB staining of the membrane as a loading control. (B) The indicated cells were analyzed by confocal microscopy in the presence of PlasMem Bright (red). Merged images of EGFP (green), PlasMem Bright, and Hoechst 33342 (blue) are shown. Scale bar, 10 µm. (C) cDNA was prepared from Ba/F3 cells, and the mRNA levels of m*Tmem63* family members were quantified by real-time RT-PCR and expressed as relative expression using the ΔCt method with 36B4 as an internal control. Triplicate samples are shown; bars indicate mean values.
